# Neutralizing and Neuraminidase Antibodies Correlate With Protection Against Influenza During a Late Season A/H3N2 Outbreak Among Unvaccinated Military Recruits

**DOI:** 10.1093/cid/ciz1198

**Published:** 2019-12-16

**Authors:** Carol D Weiss, Wei Wang, Yun Lu, Monisha Billings, Angelia Eick-Cost, Laura Couzens, Jose L Sanchez, Anthony W Hawksworth, Peter Seguin, Christopher A Myers, Richard Forshee, Maryna C Eichelberger, Michael J Cooper

**Affiliations:** 1 Office of Vaccines Research and Review, Center for Biologics Evaluation and Research, US Food and Drug Administration, Silver Spring, Maryland, USA; 2 Office of Biostatistics and Epidemiology, Center for Biologics Evaluation and Research, US Food and Drug Administration, Silver Spring, Maryland, USA; 3 Armed Forces Health Surveillance Branch, Defense Health Agency, Department of Defense, Silver Spring, Maryland, USA; 4 Operational Infectious Diseases, Naval Health Research Center, San Diego, California, USA; 5 III Marine Expeditionary Force, Okinawa, Japan

**Keywords:** influenza hemagglutinin, influenza neutralizing antibodies, neuraminidase inhibition antibodies, correlates of protection, H3N2 subtype

## Abstract

**Background:**

Antibodies that inhibit hemagglutination have long been considered a correlate of protection against influenza, but these antibodies are only a subset of potentially protective antibodies. Neutralizing and neuraminidase antibodies may also contribute to protection, but data on their associations with protection are limited.

**Methods:**

We measured preoutbreak hemagglutinin pseudovirus neutralization (PVN) and neuraminidase inhibition (NAI) antibody titers in unvaccinated military recruits who experienced an H3N2 influenza outbreak during training. We conducted a case-control study to investigate the association between titers and protection against influenza illness or H3N2-associated pneumonia using logistic regression.

**Results:**

With every 2-fold increase in PVN titer, the odds of medically attended polymerase chain reaction–confirmed H3N2 infection (H3N2^+^) decreased by 41% (odds ratio [OR], 0.59; 95% confidence interval [CI], .45 to .77; *P* < .001). Among those who were H3N2^+^, the odds for pneumonia decreased by 52% (OR, 0.48; CI, .25 to .91; *P* = .0249). With every 2-fold increase in NAI titer, the odds of medically attended H3N2 infection decreased by 32% (OR, 0.68; 95% CI, .53 to .87; *P* = .0028), but there was no association between NAI titers and H3N2-associated pneumonia. There was also no synergistic effect of PVN and NAI antibodies.

**Conclusions:**

PVN and NAI titers were independently associated with reduced risk of influenza illness. NAI titers associated with protection had greater breadth of reactivity to drifted strains than PVN titers. These findings show that PVN and NAI titers are valuable biomarkers for assessing the odds of influenza infection.

## Teaser

We investigated preexisting neutralizing and neuraminidase inhibition (NAI) antibody titers and the odds of H3N2 influenza infection during an outbreak among unvaccinated military recruits. Neutralizing and NAI titers independently decreased the odds of infection with every 2-fold increase in titer.

Due to the stressful nature of military recruit training and the close living quarters, recruits are particularly susceptible to respiratory infections [1–3]. To mitigate these factors, mandatory vaccinations, including seasonal influenza vaccines, are administered to all new and other active-duty personnel [4]. However, many incoming recruits who enter training during late spring and summer may not be vaccinated if supplies are depleted.

The Marine Corps Recruit Depot at Parris Island, South Carolina, trains recruits in 13-week cycles. Between 1 May 2016 and 28 May 28 2016, the Parris Island clinic observed a substantial increase in the number of recruits who presented with febrile respiratory illness (FRI). As part of the active surveillance for FRI among recruits [4], nasal swab specimens were tested for respiratory pathogens using reverse transcription polymerase chain reaction (RT-PCR) at the Naval Health Research Center in San Diego, California. None of the recruits at Parris Island in May 2016 had been vaccinated against influenza at the training site. Influenza vaccination history was not recorded, but anyone previously vaccinated would likely have received the vaccine many months earlier. Therefore, this outbreak presented an opportunity to identify preexisting antibodies that contribute to protection against influenza infection in healthy, young adults.

A hemagglutination inhibition (HAI) antibody titer of ≥40 has long been considered a marker or correlate of protection against influenza infection. HAI antibodies bind to the hemagglutinin (HA) protein on the surface of the virus and neutralize the virus by inhibiting attachment to sialic acid receptors on target cells. However, HAI antibodies represent a subset of antibodies that can contribute to protection against influenza. Antibodies that bind to sites on HA that are not captured in HAI assays can also neutralize virus. However, these antibodies have not been routinely measured because standard neutralization assays are impractical for running large numbers of samples. However, a high-throughput method that measures neutralizing antibody titers to seasonal influenza strains using HA pseudoviruses is now available [5]. Antibodies to neuraminidase (NA) also contribute to protection against disease but, like neutralizing antibodies, are often not measured. NA inhibition (NAI) antibody titers can be measured using an enzyme-linked lectin assay (ELLA) [6], providing a practical platform for serologic analysis of samples collected in vaccine or outbreak influenza studies.

Here, we investigated the association between preoutbreak, influenza-specific antibody titers and influenza A/H3N2 infection by comparing pseudovirus neutralizing (PVN) and NAI antibody titers in sera from influenza cases and controls. The primary analysis estimated the association of H3N2 infection with influenza-specific antibody titers from individuals diagnosed using RT-PCR testing (H3N2^+^ cases) and the control group, which consisted of individuals who visited the clinic with FRI symptoms but tested negative for H3N2 influenza, plus individuals who did not visit the clinic. Secondary analyses examined the association of PVN and NAI titers with pneumonia among H3N2^+^ cases and whether the protective effects of PVN and NAI titers against H3N2 infection were synergistic.

## METHODS

### Recruits

We studied a Parris Island training unit that included recruits who presented to the clinic with FRI symptoms in May 2016 and recruits who shared housing and training facilities with those recruits but did not present to the clinic with illness. Chest X rays were performed to diagnose pneumonia when clinically suspected. Pretraining sera samples were retrieved from the Department of Defense Serum Repository. Deidentified samples were sent to the Center for Biologics Evaluation and Research at the US Food and Drug Administration (FDA), where serology tests were performed with approval by the FDA Research in Human Subjects Committee. Recruits who received the 2015–2016 seasonal influenza vaccine between the serum sample collection date and the end of the outbreak were excluded.

**Figure 1.**
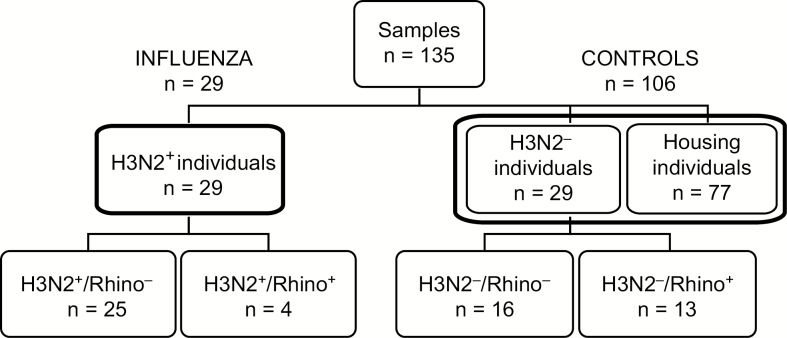
Sample selection. A total of 135 serum samples were tested; 58 sera were from individuals with febrile respiratory illness (FRI), 29 of whom tested positive by reverse transcription polymerase chain reaction (RT-PCR) for influenza A/H3N2. Of these H3N2^+^ cases, 25 were negative for other respiratory pathogens, while 4 were also positive for rhinovirus (Rhino^+^). Another 29 sera were from individuals with FRI who tested negative for influenza A/H3N2 by RT-PCR. Of these H3N2^−^ individuals, 16 were negative for all other respiratory pathogens and 13 were Rhino^+^. The H3N2^−^ (n = 29) and housing (n = 77) individuals were used as the control group (n = 106).

Sera samples from 135 subjects were analyzed. This included 58 FRI recruits and 77 housing recruits who did not report to the clinic but shared housing and training facilities with FRI cases and were likely exposed to the outbreak H3N2 virus. Among the 58 FRI recruits, 29 had RT-PCR–confirmed influenza infection (H3N2^+^), 4 of whom were also RT-PCR–confirmed for rhinovirus infection (H3N2^+^/Rhino^+^). The remaining 25 H3N2^+^ recruits tested negative by RT-PCR for rhinovirus (H3N2^+^/Rhino^−^). The other 29 FRI recruits tested negative by RT-PCR for A/H3N2 influenza (H3N2^−^), with 16 also testing negative for rhinovirus (H3N2^−^/Rhino^−^) and 13 testing positive for rhinovirus (H3N2^−^/Rhino^+^; Figure 1). Demographic information in Table 1 indicates no clear differences in age or ethnicity between groups.

**Table 1.** Demographic Information

Group	All Febrile Respiratory Illness	H3N2^+^		H3N2^−^		Housing	H3N2^+^/Pneumonia		H3N2^−^/Pneumonia	
		Rhino^−^	Rhino^+^	Rhino^−^	Rhino^**+**^		Rhino^−^	Rhino^+^	Rhino^−^	Rhino^+^
Individuals	n = 58	n = 25	n = 4	n = 16	n = 13	n = 77	n = 9	n = 3	n = 12	n = 12
Age in years, median	17–26, 19	17–26, 19	20–21, 20	18–24, 19	17–24, 21	17–31, 19	18–25, 19	18–24, 20	18–24, 19	17–24, 21
Female	9 (16)	5 (20)	0	3 (19)	1 (8)	0	2 (22)	0	1 (8)	1 (8)
White	30 (52)	14 (56)	1 (25)	6 (37)	9 (69)	45 (58)	3 (33)	1 (33)	5 (42)	8 (67)
Black	9 (16)	3 (12)	2 (50)	3 (19)	1 (8)	11 (14)	3 (33)	2 (67)	3 (25)	1 (8)
Hispanic	15 (26)	7 (28)	1 (25)	5 (31)	2 (15)	17 (22)	2 (22)	0	2 (17)	2 (17)
Asian	2 (3)	0	0	2 (13)	0	2 (3)	0	0	2 (17)	0
Unknown	2 (3)	1 (4)	0	0	1 (8)	2 (3)	1 (11)	0	0	1 (8)

H3N2^+^ tested positive by reverse transcription polymerase chain reaction (RT-PCR) for A/H3N2 influenza. H3N2^−^ tested negative by RT-PCR for A/H3N2 influenza. Rhino^+^ tested positive by RT-PCR for rhinovirus. Rhino^−^ tested negative by RT-PCR for rhinovirus. Pneumonia confirmed by chest X ray when clinically indicated. Percent of individuals in a group is shown in parentheses.

### Virological Testing

Nasal swabs were tested using RT-PCR for respiratory pathogens that included adenovirus, rhinoviruses, respiratory syncytial virus, and influenza A and B viruses. FRI recruits who tested positive for H3N2 by RT-PCR were considered H3N2^+^ cases.

### Influenza Viruses Used in Serological Studies

The 2015–2016 season influenza vaccine included A/Switzerland/9715293/2013 (H3N2) from the genetic group 3C.3a. The HA and NA genes from the outbreak virus were in the same genetic group but differed at 2 HA residues, causing a loss of glycosylation at residue 45. The NA from the outbreak strain differed in multiple residues compared with the vaccine strain, but ferret antisera did not detect antigenic differences (data not shown). Serologic assays measured antibody titers against the homologous A/H3N2 outbreak virus, A/South Carolina/NHRC 75458/2016 (SC16), as well as the following heterologous H3N2 viruses that were recommended in past vaccines: A/Moscow/20/99 (H3N2) (MW99), A/Wyoming/03/03 (H3N2) (WY03), A/Texas/50/12 (H3N2) (TX12), and the heterosubtypic H1N1 vaccine component, A/California/7/2009 (H1N1) (CA09).

### Serological Analyses

Serum-neutralizing and NA-inhibiting antibody titers were measured in PVN and ELLA assays. HA pseudoviruses that bore HAs that corresponded to strains of interest were used in neutralization assays as previously described [5]. The serum dilution that caused a 95% or 50% reduction of relative luminescence units (RLUs) compared with the control without serum (IC_95_ or IC_50_ neutralizing antibody titer, respectively) was used as the neutralization end point titer. Titers that measured below the lowest serum dilution of 1:40 were reported as 20 for statistical analysis. The mean titer from at least 2 independent experiments was reported.

The NAI antibody titer was performed as previously described [6] using HA from an H6 avian virus to avoid assay interference by HAI antibodies. NAI titers were determined by nonlinear regression of the percent inhibition of NA activity. The inverse of the dilution that resulted in 50% or 95% NA activity was reported as the IC_50_ or IC_95_, respectively, with the lowest titer of <10 (the lowest dilution in the assay was 1:10) reported as 5 for statistical analysis. The geometric mean titer of duplicate tests was reported.

### Study Design and Statistical Analyses

This retrospective, case-control study involved H3N2^+^ FRI individuals and controls that consisted of H3N2^−^ FRI individuals and individuals who did not visit the clinic but shared facilities with those who were H3N2^+^. Our prespecified, statistical analyses plan combined the 77 housing and 29 H3N2^−^ individuals as controls (total 106; Figure 1) to improve power for assessing the relationship between titers and H3N2 infection.

The primary analysis tested whether RT-PCR–confirmed H3N2 influenza infection was associated with NAI or PVN antibody titers for the homologous A/H3N2 SC16 antigen. Univariate logistic regression was conducted, and the binary H3N2^+^ status was used as the outcome variable. Log2 transformed antibody titers were used in all analyses. IC_95_ PVN or IC_50_ NAI titers were used as the explanatory variable. The change in odds of H3N2 influenza infection associated with a 2-fold increase in antibody titer was estimated. To test the impact of antibody specificity on the association, similar logistic regressions were conducted for heterologous antigens from past A/H3N2 viruses TX12, WY03, and MSC99 and the A/H1N1 virus CA09.

The hypothesis that NAI and PVN antibody titers for the homologous antigen work in synergy to protect against RT-PCR–confirmed influenza infection was also tested by conducting a multivariate logistic regression that included the main effects of both IC_95_ PVN titers and IC_50_ NAI titers and their interaction term. Another secondary analysis tested whether pneumonia was associated with NAI or PVN antibody titers among RT-PCR–confirmed H3N2^+^ cases using univariate logistic regression.

To exclude the possibility that our control group included individuals who had false-negative H3N2 RT-PCR results due to poor timing of nasal sample collection, we conducted a sensitivity analysis using only the housing group as the control. To determine whether the method used to determine antibody titer would impact the association of titer with H3N2 infection, another sensitivity analysis was performed using IC_50_ PVN titers (instead of IC_95_ PVN titers) or IC_95_ NAI titers (instead of IC_50_ NAI titers).

We used statistical software R 3.5.1 (R Foundation for Statistical Computing, Vienna, Austria) for our analysis, with a *P* value of <.05 considered statistically significant. 

## RESULTS

### Distribution of PVN and NAI Titers Against the Outbreak Virus

**Figure 2.**
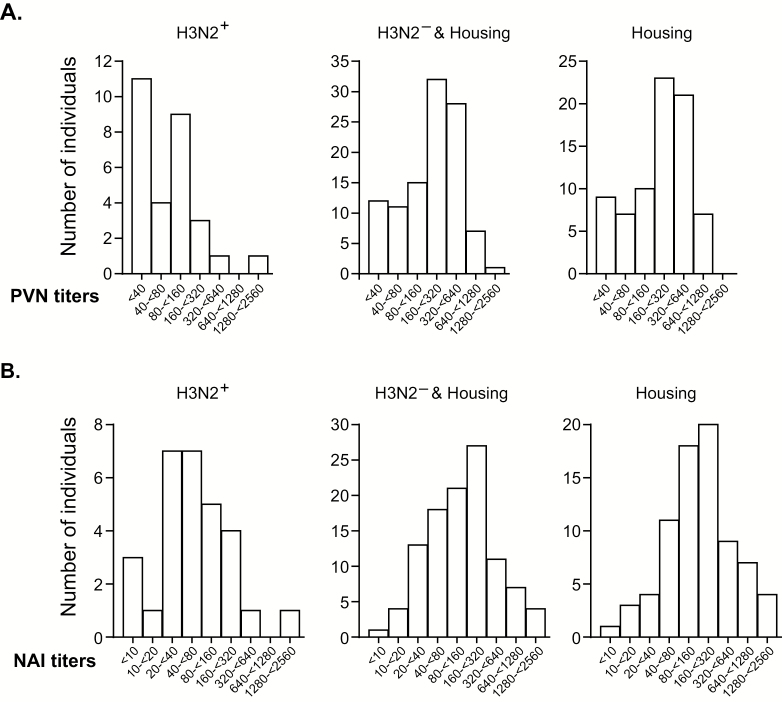
Distribution of antibody titers. PVN IC_95_ titers (*A*) and NAI IC_50_ titers (*B*) for individuals who tested positive (H3N2^+^) or negative (H3N2^−^) for H3N2 by polymerase chain reaction and housing controls, analyzed combined and separately. Bars show the number of individuals with titers indicted on the *x*-axis. IC_95_ and IC_50_ are the antibody titers required to inhibit 95% or 50% percent of the input infectivity for PVN or NAI activity for NAI. Abbreviations: NAI, neuraminidase inhibition; PVN, pseudovirus neutralization.

First, we examined the number of individuals in each group with a specific PVN or NAI titer range against the outbreak SC16 strain (Figure 2). Compared with the H3N2^+^ group, the H3N2^−^ and housing control group, as well as the housing group alone, had overall higher titers. The H3N2^+^ group had a PVN geometric mean titer (GMT) of 65.48 (95% confidence interval [CI], 42.48 to 100.9), while the H3N2^−^ and housing control group had a PVN GMT of 181.4 (95% CI, 146.6 to 224.5), and the housing group had a PVN GMT of 189.1 (95% CI, 146.5 to 244.1). Among the H3N2^+^ group, the greatest proportion of individuals had titers <160. In contrast, among the H3N2^−^ and housing control group and the housing group alone, most individuals had titers >160 (Figure 2A). A similar shift in the distribution of titers was seen for NAI titers when titers from the H3N2^+^ group were compared with the combined H3N2^−^ and housing control group or housing group alone (Figure 2B). The H3N2^+^ group had an NAI GMT of 59.46 (95% CI, 36.55 to 96.72), while the H3N2^−^ and housing control group had an NAI GMT of 135.1 (95% CI, 107.3 to 170.2), and the housing group had an NAI GMT of 167.0 (95% CI, 127.2 to 219.2).

**Figure 3.**
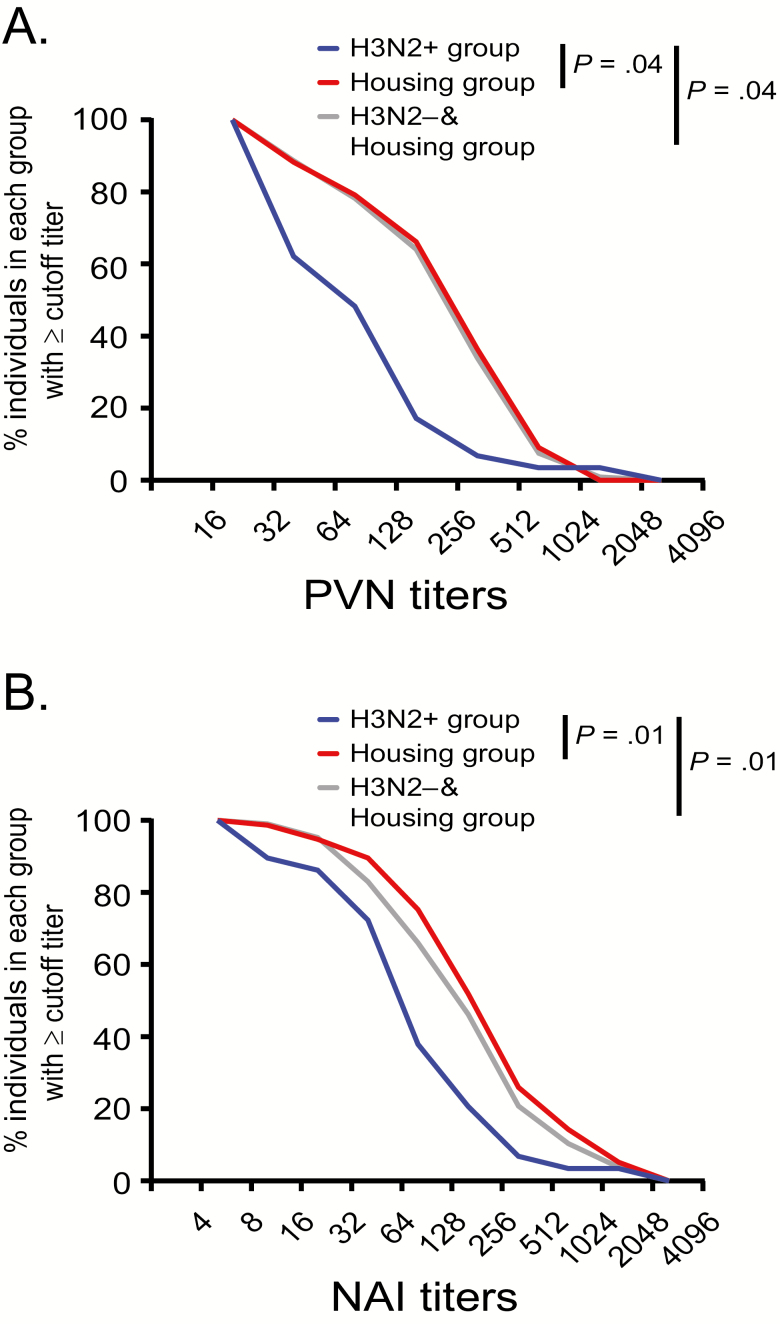
Reverse cumulative distribution curves. The percent of individuals with (*A*) PVN IC_95_ and (*B*) NAI IC_50_ titers less than or equal to the shown titers is plotted for the H3N2^+^ (blue curve), housing (red curve), and H3N2^−^ and housing control (light gray curve) groups. Differences between the groups and titers were analyzed using 2-way analysis of variance. *P* values <.05 were considered significant. Abbreviations: NAI, neuraminidase inhibition; PVN, pseudovirus neutralization.

Likewise, when PVN titers are presented as reverse cumulative distribution (RCD) curves, the curves for the H3N2^+^ group are shifted toward lower titers compared with the H3N2^−^ and housing control group or the housing group alone (Figure 3A). The RCD curves for NAI titers are similarly shifted toward lower titers for the H3N2^+^ group compared with the combined H3N2^−^ and housing control group (Figure 3B).

### Association of Neutralizing and NA Inhibition Antibody Titers With Influenza A/H3N2 Infection 

**Figure 4.**
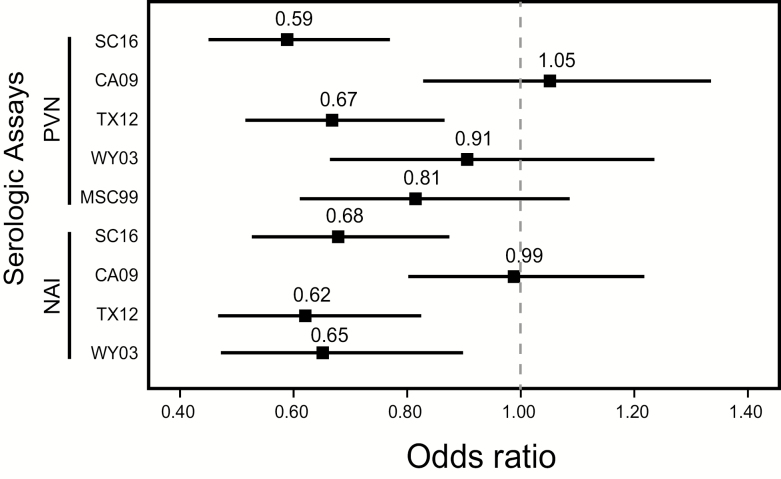
Serologic correlates against polymerase chain reaction–confirmed influenza infection. Odds ratios were determined by analysis of PVN IC_95_ and NAI IC_50_ antibody titers associated with H3N2^+^ cases and controls. Stata 15 was used to generate this figure. Abbreviations: for the pseudoviruses corresponding to the following strains: SC16 = A/South Carolina/NHRC 75458/2016 (H3N2); MW99 = A/Moscow/20/99 (H3N2); WY03 = A/Wyoming/03/03 (H3N2); TX12 = A/Texas/50/12 (H3N2); and CA09 = A/California/7/2009 (H1N1); NAI, neuraminidase inhibition; PVN, pseudovirus neutralization.

Next, we estimated the change in odds of being H3N2^+^ at increasing serum antibody titers. Using the H3N2^−^ and housing control group, logistic regression analysis indicated that the odds of H3N2^+^ infection decreased by 41% (odds ratio [OR], 0.59; 95% CI, .45 to .77; *P* = .0001) with every 2-fold increase in PVN titer against the HA of the outbreak H3N2 SC16 virus (Table 2, Figure 4). In contrast, there was no statistically significant association between H3N2 influenza infection and PVN titers against CA09 (H1N1), confirming the specificity of the correlation between PVN titers and the outbreak H3N2 virus. Increasing NAI titers were also associated with a decrease in the odds of PCR-confirmed H3N2 infection (Table 2, Figure 4). The odds of H3N2^+^ infection decreased by 32% (OR, 0.68; 95% CI, .53 to .87; *P* = .003) with every 2-fold increase in NAI titer against the H3N2 SC16 virus.

**Table 2.** Association of Pseudovirus Neutralization and Neuraminidase Inhibition Antibody Titers With Risk of H3N2 Infection

Influenza Cases	Control Group	Antigen	OR for PVN IC_95_ Titers			OR for NAI IC_50_ Titers		
			**OR**	**95% CI**	***P* Value**	**OR**	**95% CI**	***P* Value**
H3N2^+^, n = 29	H3N2^−^ and housing controls, n = 106	SC16	0.59	.45–.77	.0001	**0.68**	.53–.87	.0028
		TX12	0.67	.52–.87	.0023	**0.62**	.47–.82	.001
		WY03	**0.91**	.66–1.24	.532	**0.65**	.47–.90	.009
		MSC99	**0.81**	.61–1.09	.162	Not done		
		CA09 (H1N1)	**1.05**	.83–1.34	**.681**	**0.99**	.80–1.22	.91
H3N2^+^, n = 29	H3N2^−^, n = 29	SC16	**0.60**	.41–.86	.0059	**0.87**	.63–1.20	.391
H3N2^+^, n = 29	Housing controls, n = 77	SC16	**0.58**	.44–.77	**.0002**	**0.62**	.47–.82	.0007
H3N2^+^ and pneumonia, n = 12	H3N2^+^, no pneumonia, n = 17	SC16	**0.48**	.25–.91	.0249	**1.12**	**.74–1.69**	.597
**Influenza Cases**	**Control Group**	**Antigen**	**OR for Alternative PVN IC**_**50**_ **Titers**			**OR for Alternative NAI IC**_**95**_ **Titers**		
			**OR**	**95% CI**	***P* Value**	**OR**	**95% CI**	***P* Value**
H3N2^+^, n = 29	H3N2^−^ and housing controls, n = 106	SC16	**0.68**	.51–.90	.0074	**0.67**	.49–.93	.0175

A *P* value <.05 is considered significant. H3N2^+^ tested positive by reverse transcription polymerase chain reaction (RT-PCR) for A/H3N2 influenza. H3N2^−^ tested negative by RT-PCR for A/H3N2 influenza. Pneumonia confirmed by chest X ray when clinically indicated. Virus antigens: SC16 = A/South Carolina/NHRC 75458/2016 (H3N2); MW99 = A/Moscow/20/99 (H3N2); WY03 = A/Wyoming/03/03 (H3N2); TX12 = A/Texas/50/12 (H3N2); and CA09 = A/California/7/2009 (H1N1).

Abbreviations: CI, confidence interval; IC_50_, titer that inhibited 50% of infection by the antigen tested for PVN or NA activity for NAI; IC_95_, titer that inhibited 95% of infection by the antigen tested for PVN or NA activity for NAI; NAI, neuraminidase inhibition; OR, odds ratio; PVN, pseudovirus neutralization.

We performed a sensitivity analysis to compare the standard method of reporting titers previously developed for each assay, that is, IC_95_ for PVN and IC_50_ for NAI, with alternate PVN IC_50_ and NAI IC_95_ titers (Table 2). The alternate PVN IC_50_ titer was less sensitive than the PVN IC_95_ titer; the odds of PCR-confirmed H3N2 influenza infection decreased by 32% (OR, 0.68; 95% CI, .51 to .90; *P* = .007) with every 2-fold increase in PVN IC_50_ titers. NAI IC_95_ titers indicated a 33% decrease (OR, 0.67; 95% CI, .49 to .93; *P* = .0175) in the odds of PCR-confirmed H3N2 influenza infection with every 2-fold increase in titer, similar to NAI IC_50_ titers. All subsequent analyses therefore used IC_95_ for PVN and IC_50_ for NAI.

We explored the breadth of PVN and NAI antibody reactivity associated with reduced odds of infection using titers against heterologous H3N2 antigens that circulated during each individual’s lifetime: TX12, a clade 3C virus that was the predominant clade from 2012–2015; WY03, a “Fujian-like” virus that emerged in 2002; and MW99, a virus that circulated prior to the emergence of A/Fujian-like H3N2 viruses. The odds of influenza infection during this 2016 outbreak decreased by 33% (OR, 0.67; 95% CI, .52 to .87) with every 2-fold increase in PVN titer against the prior season H3N2 TX12 virus, but the association against more distant H3N2 WY03 and MW99 viruses was not significant (Table 2 and Figure 4). For NAI titers, the risk of infection decreased 38% with increasing antibody titer (OR, 0.62; 95% CI, .47 to .82; *P* = .001). Unlike HA-specific antibodies, NAI-specific antibodies against WY03 appeared to contribute to protection, with a 35% reduction in the association between NAI titers and H3N2^+^ infection (OR, 0.65; 95% CI, .47 to .90; *P* = .009). These findings suggest that the antibodies target conserved NA epitopes. There was no association between the odds of H3N2 infection and PVN or NAI titers against CA09, an H1N1 virus, demonstrating the specificity of the association between H3N2-specific antibodies and AH3N2 infection during this outbreak.

Pearson correlation results indicated a weak, positive correlation between PVN and NAI titers for SC16 (г = 0.27). However, logistic regression that included an interaction term for SC16-specific PVN and NAI titers indicated no statistically significant synergistic effect of these different antibody specificities that may have contributed to resistance against H3N2^+^ infection (OR, 0.92; 95% CI, .78 to 1.08; *P* = .319).

### Association of Neutralizing Antibody Titers With Protection From Pneumonia

We further examined the hypothesis that the odds of a pneumonia diagnosis in H3N2^+^ cases are associated with PVN and NAI antibody titers (Table 2). Of the 29 H3N2^+^ cases, 12 were diagnosed with pneumonia. The odds of pneumonia among H3N2^+^ cases decreased by 52% with every 2-fold increase in PVN titer against H3N2 SC16 (OR, 0.48; 95% CI, .25 to .91; *P* = .025). There was no significant relationship between NAI titers and the odds of pneumonia in H3N2^+^ cases (OR, 1.12; 95% CI, .74 to 1.69; *P* = .597).

## DISCUSSION

We investigated the association between preoutbreak, influenza-specific antibody titers and influenza infection and influenza-associated pneumonia in healthy young adults at high risk of influenza H3N2 exposure in a military recruit setting. The primary analyses using H3N2^+^ cases and controls (H3N2^−^ and housing recruits) indicated a 41% and 32% reduction in the odds of testing positive for H3N2 with every 2-fold increase in PVN and NAI antibody titers, respectively. Two-fold increases in PVN, but not NAI titers, were also associated with 52% reduced odds of influenza-associated pneumonia.

The PVN and NAI titers independently correlated with protection and were not synergistic. Others have similarly shown the independence of HA-specific and NAI antibodies in protecting against influenza in various settings [7–9]. However, the levels of antibodies needed for protection against influenza illness are likely context dependent. Many factors, including age, background immunity, virus strain, and exposure intensity, can affect clinical outcomes [10]. Moreover, immune measures are highly dependent on the assay [11].

Because antibody titers reflect prior infection and vaccination history, we also investigated how antibody specificity affected the odds of influenza infection or pneumonia. We measured antibody titers to an H3N2 virus that circulated in recent years (TX12) and 2 H3N2 viruses, WY03 and MSC99, that circulated in past years (Figure 4). PVN titers were significantly associated only with the recent past H3N2 strain, although there was a trend to an association with more remote H3N2 strains. Interestingly, PVN antibody titers against WY03 were often higher than against SC16 (data not shown), suggesting that most recruits had been exposed to WY03-like viruses when they were young. High titers to the older strain may be due to “back-boosting” (recall of immune memory) during subsequent infections. However, the lack of strong association between the WY03 antibody titers and resistance against SC16 suggests that these antibodies conferred little benefit during the outbreak. As expected, there was no association with the heterosubtypic H1N1 strain. NAI titers similarly correlated with the degree of antigenic relatedness of NA among the strains tested [12]. The greater breadth of NAI antibodies associated with reduced odds of influenza supports inclusion of NA in next-generation vaccines aimed at better protection against drifted strains.

The PVN assay detects antibodies to the variable head and more conserved stem regions of HA. The strong association of PVN titers with only the outbreak and recent past, but not the more remote H3N2 strains, suggests that the protective neutralizing antibodies are predominantly focused on variable regions in the HA head. To evaluate the presence of neutralizing antibodies to conserved epitopes in the HA stem, we assessed neutralizing activity against an HA from a noncirculating, group 2 influenza A virus. We found that all sera lacked neutralizing activity against HA pseudoviruses that corresponded to an H7N2 (data not shown), further supporting the dominance of antibodies targeting the HA head.

Our study involving recruits is likely relevant to other situations that involve influenza transmissions in close quarters. Limitations of our analyses include potential false negatives in the control group due to asymptomatic H3N2 infections, symptomatic recruits not reporting to the clinic to avoid interruptions in basic training, and/or inadequate timing or collection of samples for PCR testing. These limitations, however, would only diminish statistical power of our analyses and bias the results towards the null. The coincident rhinovirus outbreak also may have affected influenza spread or symptoms in unknown ways. Other factors that we were not able to study include age, comorbid conditions, and vaccine-induced immunity.

In summary, our results show that PVN and NAI titers are valuable biomarkers for assessing the odds of influenza infection and disease. PVN and NAI titers may also prove useful for evaluating next-generation influenza vaccines. Finally, these findings support immunization of new recruits against influenza when training commences, regardless of the time of year.

## Notes

***Acknowledgments.*** The authors thank Daniel Edgeworth (Naval Health Research Center, Parris Island, SC) for gathering information on the recruits and Zheng Hu (Armed Forces Health Surveillance Branch, Health Affairs, Department of Defense) for help with the database. The authors also thank St. Jude Children’s Research Hospital for plasmids used to generate reassortant viruses used in enzyme-linked lectin assay and the US Food and Drug Administration (FDA; Silver Spring, MD) colleagues Matthew Sandbulte and Jin Gao for preparing H6N1 and H6N2 reassortant viruses and Hector Izurieta and Sarah Browne for critical reading of the manuscript.

***Financial support.*** This work was supported in part by institutional research funds from the US FDA and by an interagency award from the Armed Forces Health Surveillance Branch/GEIS of the Department of Defense (to C. W. and M. E.).

***Potential conflicts of interest.*** The authors: No reported conflicts of interest. All authors have submitted the ICMJE Form for Disclosure of Potential Conflicts of Interest.
